# Participatory Surveillance for COVID-19 Trend Detection in Brazil: Cross-sectional Study

**DOI:** 10.2196/44517

**Published:** 2023-04-26

**Authors:** Salome Wittwer, Daniela Paolotti, Guilherme Lichand, Onicio Leal Neto

**Affiliations:** 1 Department of Economics University of Zurich Zurich Switzerland; 2 Data Science for Social Impact and Sustainability ISI Foundation Turin Italy; 3 Institute for Information Security Department of Computer Science ETH Zürich Zurich Switzerland

**Keywords:** participatory surveillance, COVID-19, digital epidemiology, coronavirus, infectious disease, epidemic, pandemic, SARS-CoV-2, forecast, trend, reporting, self-report, surveillance

## Abstract

**Background:**

The ongoing COVID-19 pandemic has emphasized the necessity of a well-functioning surveillance system to detect and mitigate disease outbreaks. Traditional surveillance (TS) usually relies on health care providers and generally suffers from reporting lags that prevent immediate response plans. Participatory surveillance (PS), an innovative digital approach whereby individuals voluntarily monitor and report on their own health status via web-based surveys, has emerged in the past decade to complement traditional data collection approaches.

**Objective:**

This study compared novel PS data on COVID-19 infection rates across 9 Brazilian cities with official TS data to examine the opportunities and challenges of using PS data, and the potential advantages of combining the 2 approaches.

**Methods:**

The TS data for Brazil are publicly accessible on GitHub. The PS data were collected through the Brazil Sem Corona platform, a Colab platform. To gather information on an individual’s health status, each participant was asked to fill out a daily questionnaire on symptoms and exposure in the Colab app.

**Results:**

We found that high participation rates are key for PS data to adequately mirror TS infection rates. Where participation was high, we documented a significant trend correlation between lagged PS data and TS infection rates, suggesting that PS data could be used for early detection. In our data, forecasting models integrating both approaches increased accuracy up to 3% relative to a 14-day forecast model based exclusively on TS data. Furthermore, we showed that PS data captured a population that significantly differed from a traditional observation.

**Conclusions:**

In the traditional system, the new recorded COVID-19 cases per day are aggregated based on positive laboratory-confirmed tests. In contrast, PS data show a significant share of reports categorized as potential COVID-19 cases that are not laboratory confirmed. Quantifying the economic value of PS system implementation remains difficult. However, scarce public funds and persisting constraints to the TS system provide motivation for a PS system, making it an important avenue for future research. The decision to set up a PS system requires careful evaluation of its expected benefits, relative to the costs of setting up platforms and incentivizing engagement to increase both coverage and consistent reporting over time. The ability to compute such economic tradeoffs might be key to have PS become a more integral part of policy toolkits moving forward. These results corroborate previous studies when it comes to the benefits of an integrated and comprehensive surveillance system, and shed light on its limitations and on the need for additional research to improve future implementations of PS platforms.

## Introduction

The global COVID-19 pandemic in March 2020 has had unprecedented consequences around the world. It caused widespread illness and deaths, as well as worldwide economic, political, and social repercussions [1]. The occurrence of such an extraordinary event emphasizes the need for well-functioning disease surveillance systems to detect and monitor disease outbreaks and epidemics. Countries use disease monitoring systems to assess, predict, and mitigate infectious disease outbreaks [2,3]. Reliable and timely data are critical to protect populations and build the foundation for governments, policy makers, and officials to intervene and prevent widespread infections [4]. As such, developing and improving on existing surveillance methods remains a rapidly growing and emerging field [5].

Most current surveillance systems, listed here as traditional surveillance (TS) systems, rely largely on traditional health care institutions, such as clinics, hospitals, and laboratories, to systematically collect data from practitioners as a public health monitoring tool [2]. Health care providers send reports to public health officials with certain regional or national data aggregation. In a few cases (usually, only a small percentage), these reports are then confirmed by laboratory analysis. Reported cases are then accounted as official disease cases [6-8]. Since the data are sourced from different institutions, aggregation often involves several time lags throughout the chain of data collection, reducing the timeliness of measures and actions [4,9,10]. Moreover, the true burden of the disease is often underestimated. In industrialized countries, healthy adults with no previous conditions usually do not visit a doctor if their symptoms remain mild. In emerging countries, socioeconomically weak communities lack access or financial resources to seek medical aid and thus are overlooked by TS [11]. Over the last 2 decades, new digital disease surveillance approaches have emerged to supplement traditional data collection, such as participatory surveillance (PS). Participatory disease surveillance is understood as an approach that directly engages the public in providing health data. Individuals monitor and assess their own health status and are encouraged to submit self-reports through digital platforms using mobile apps, websites, and phone-based surveys via SMS text messages or automated calls (interactive voice response [IVR]) [12]. Any individual can register on the platform (if they live in a country where a PS platform is deployed) and participate on a voluntary basis. As such, digital crowdsourced data can be aggregated and analyzed in large numbers [13-15]. Users are asked to regularly complete a questionnaire aimed at gathering information about their current (lack of) symptoms, access to health care, risk exposure, and medications. In the case of COVID-19, the main difference compared to TS is the lack of laboratory-confirmed testing; positive cases are categorized as such only based on the reporting of certain symptoms, with a so-called syndromic surveillance approach. The added value of PS systems is that they can even reach individuals who do not engage with health care providers because of financial or cultural factors, because they live too remotely to have access to health care facilities, or because their symptoms are too mild to cause concern [12]. The rapid global increase in the use of mobile phones and wide access to the internet are largely responsible for the rise of such digital surveillance systems [3]. By integrating an additional subset of the population not covered by TS, the complementary data provide an additional layer of surveillance, potentially enabling more accurate detection of ongoing infections as well as anticipation of trend changes [13,14].

PS systems have so far proven to be accurate and reliable for influenza-like illness (ILI) surveillance [15,16]. Most recently, as testing capacities were exhausted across many countries in the context of the pandemic, PS systems were implemented to support traditional systems in monitoring and controlling COVID-19 infections [17,18]. So far, Brazil is the first and only Latin American country that has implemented a PS system on a large scale to carry out syndromic surveillance, specifically during the 2014 FIFA World Cup [19] and the 2016 Olympic Games [20]. Lately, a local Brazilian health authority has used a PS platform to complement the traditional system, with the goal of optimizing the targeting of test areas during the COVID-19 pandemic [17]. This PS system has been shown to be beneficial in identifying risk clusters for infections in this context; in particular, it was able to cover blind spots of the TS system, showcasing the potential to increase its sensitivity by complementing it with additional data, and to allocate scarce resources more efficiently by prioritizing certain areas for the distribution of test kits. Notably, in Europe and the United States, health agencies and governments are increasingly prone to using the innovative and digital PS approach as a complementary source of disease surveillance.

Low public funding and suboptimal resource allocation persist in the Brazilian health sector [21]. The limitations of the TS system stress the need to reduce the burden of diseases among vulnerable and socioeconomically weak communities. This motivates the study to examine the opportunities and challenges of a PS system in the context of COVID-19 case detection across 9 Brazilian cities. During 7 months of the global pandemic in 2020, several city-level governments implemented the *Brazil Sem Corona* PS platform to gather additional insights on the spread of the disease. *Brazil Sem Corona* was an initiative led by Colab in partnership with several local governments from Brazilian cities, with the purpose of leveraging PS data to complement TS systems in order to mitigate COVID-19 risk at the local level. For our analysis, we focused on 9 cities with the largest PS participation across Brazil. Our objective was to investigate the capability of PS data to approximately mirror traditional infection rates captured through TS, as well as the relevance of citizen participation for the identification of trends in COVID-19 cases. Furthermore, we investigated the potential benefits of combining the PS and TS systems for forecasting case trends. Among the benefits, we showed that the PS system captures a part of the population that is so far overseen by traditional sources.

## Methods

### Goal

In this work, the goal was to compare daily official COVID-19 infections at the municipality level with daily PS infection numbers.

### TS Data

TS data for Brazil are publicly accessible on GitHub [22]. These data aggregate the official laboratory-confirmed daily new COVID-19 cases at the municipality level. Using the 2020 population size estimates for each city [23], we calculated the infection rate per 100,000 inhabitants as follows:



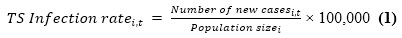



where *i* denotes the city and *t* denotes the day.

### PS Data

PS data for this study were collected through the *Brazil Sem Corona* platform, a Colab participatory platform developed previously [24]. To gather information on an individual’s health status, each participant was asked to fill out a daily questionnaire in the Colab app on symptoms as well as exposure. The app was available on the Apple Store as well as the Google Play Store. The list of symptoms was based on the COVID-19 case definition and contained the following: fever, cough, shortness of breath, runny nose, sore throat, headache, fatigue, nausea, rash, joint pain, chills, diarrhea, and loss of taste. Additionally, participants were asked to report about medication intake and whether they sought a health care facility for their symptoms. For this study, Colab subsequently provided access to the anonymized data set of *Brazil Sem Corona*.

### Ethical Considerations

Before filling out the questionnaire, participants were asked to agree with an informed consent form in the registration phase. The form described the study and the purpose of the project, and provided information about how the data could be used by third parties for research analysis purposes. Access to the data and study was approved by the Colab Institutional Management Board. All methods were carried out in accordance with guidelines and regulations, including but not limited to the *Lei Geral da Proteção de Dados – LGPD*, the official regulation on data privacy and protection valid in Brazil. All collected study data were anonymous and deidentified. No compensation was provided to the participants. The data used in this study can be made available through a formal request to the Colab team.

### Data Collection

The platform was set up online on March 20, 2020, right after the World Health Organization officially declared COVID-19 as a public health issue with pandemic implications on March 11, 2020 [25]. During the period following this official statement, Brazilian newspapers and magazines extensively advertised the *Brazil Sem Corona* platform, leading to an increase in self-report submissions [26-28].

The increase in the number of participants allowed the creation of a database of symptom-based reports that could be used for the analysis. Each report was categorized into one of the following categories: (1) no symptoms, (2) light symptoms, (3) suspected COVID-19 case, (4) severe suspected COVID-19 case, and (5) confirmed case. In order to be categorized as a suspected case, the user must report fever together with at least one other symptom. If along with these symptoms either medication intake is reported or a health care facility is sought, the report is labeled as severe suspected case [29]. Only those users reporting a positive laboratory COVID-19 test result were categorized as confirmed cases. All the suspected, severe suspected, and confirmed cases were then treated as *possible COVID-19 cases*, while light and no symptom cases were treated as *negative cases*. The inclusion of not only confirmed cases but also suspected cases is one of the key differences with respect to case counts performed in TS settings.

For each city in which data were collected, we aggregated the reports submitted for each day and calculated the daily infection rate:



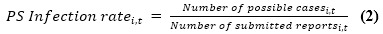



where *i* denotes the city and *t* denotes the day.

Data sparsity and fluctuations were addressed by applying a simple but powerful tool called LOESS to the PS data. It fits smooth lines to empirical data using a nonparametric approach [30]. For all 9 cities, the smoothed PS and TS infection rates were then compared.

### Pearson Correlation Calculation

To measure the statistical relationship between the 2 time series, we calculated the Pearson correlation (*PC_t_*) coefficient for the original time series. Additionally, we used a 7-day and 14-day lagged PS series to calculate *PC_t-_*_7_ and *PC_t-_*_14_. Finally, we determined the coefficients over a reduced 4-month observation period during which public engagement and participation rates were the largest.

Finally, we looked at the proportion among self-reported cases of confirmed cases and the percentage of confirmed cases seeking health care assistance.

### Forecasting Models

**Figure 1:**
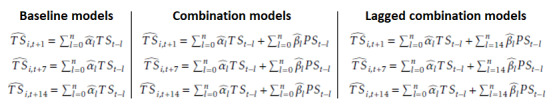
Model overview. 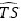 stands for the estimation, whereas TS stands for the true value. By weighing n past components, 1-, 7-, and 14-day forecasts are estimated. The model parameters 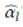 and 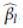 are estimated based on a training subsample that contains the first 80% of the data. The out-of-sample forecasting accuracy is then calculated based on the remaining 20% of the data. Thus, the first 80% of the observation period is used to fit the models, while the remaining 20% is used to evaluate its performance. PS: participatory surveillance; TS: traditional surveillance.

In order to further assess the added value of insights generated by PS systems, we used the PS data to inform 3 different forecasting models to predict incidence rates for COVID-19 with a time horizon varying between 1 and 3 weeks (Figure 1). TS data were used to generate a baseline model that represents the “ground truth” against which the forecasting models were compared.

Thus, the first model (further referred to as the *baseline model*) was a univariate model based on only TS data. The second and third models (referred to as the *combination model* and *lagged combination model*, respectively) were bivariate models integrating both TS and PS data. The third model also used a 14-day lag in the PS incidence rate series. For all 3 models, we used a linear autoregression function with *n* daily lagged components. For each city, the optimal number of independent variables was selected based on the Akaike Information Criterion (AIC) that estimates the prediction error and quality of statistical models. Thereby, the explained part of the variation was maximized while using only the lowest possible amount of time lag.

To evaluate the performance of the forecasting models, we calculated the root mean squared error (RMSE) and mean absolute error (MAE). The RMSE measures the difference between predicted and true values as follows:



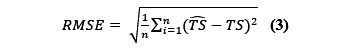



The MAE measures the average of the absolute errors between predicted and true values as follows:



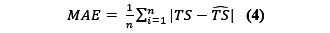



## Results

Reports were collected between March 20, 2020, and October 20, 2020. The analysis covered the whole 7-month observation period. Even though the platform was accessible all around Brazil, nearly 65% of the reports were submitted from 9 cities. Therefore, the study focused only on those cities with the largest number of submitted reports. People from Teresina, Caruaru, Santo Andre, Niteroi, Recife, Porto Alegre, Campinas, Sao Paulo, and Rio de Janeiro submitted an aggregated total of 83,005 reports (13,582 individuals).

To assess the importance of participation, we compared the capability of the PS data to mirror the infection rates reported by the TS systems implemented in the 9 cities. In Table 1, we present the 9 cities with the largest PS participation (the highest number of submitted reports across Brazil). As the population size varied between the cities, we additionally weighted the number of submitted reports by the population size displayed by the variable “number of reports by 100.” As participants could report several times throughout the observation period, there was a relatively large number of submitted reports compared with the number of participants. Even though São Paulo recorded a relatively large number of submitted reports, it was ranked only second to last due to its large population size. Teresina, Caruaru, and Santo André were ranked as the top 3 based on the variable “number of reports by 100.” The large participation in those 3 cities was not coincidental but rather driven by massive local media campaigns led by local officials [26-28]. Those social media campaigns seem to have impacted the participation behavior, underlining the government’s role. Additionally, we displayed the variable “share of zeros,” which captures the share of observation days on which no possible COVID-19 case was recorded on the platform within a city. This problem is often referred to as *zero inflation* (as the sample contains an excess of zeros) and is equivalent to censored data. Those numbers were negatively correlated with the level of engagement, as the cities at the bottom of the ranking displayed a striking share of zeros (as high as 96%).

**Figure 2:**
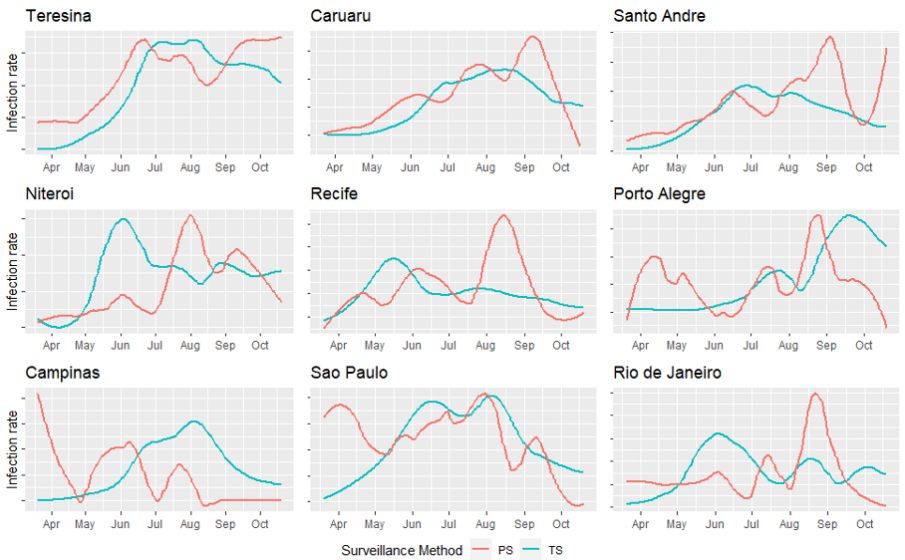
Daily PS and TS infection rates per city. The graphs are arranged according to the weighted participation from top left to bottom right. PS: participatory surveillance; TS: traditional surveillance.

As mentioned in the Methods section, every submitted report was categorized as either a negative COVID-19 case or suspected COVID-19 case based on the symptoms reported in the survey. In Figure 2, we show the TS and PS incidence rates over the entire 7-month observation period to compare differences in detected infection rates between the 2 surveillance methods. The 9 cities have been arranged according to the participation ranking (Table 1) from top left to bottom right. The top row displays the 3 cities with the largest community engagement (Teresina, Caruaru, and Santo André). Here, infections identified through PS and TS followed a relatively similar pattern, at least between March and August 2020. For most cities, the trend of detected cases diverged between the 2 methods toward the end of the observation period, which can be explained by the relatively low number of submitted reports over that interval. More details on the daily number of submitted reports can be found in Figure S1 in Multimedia Appendix 1. From the graphical analysis, it follows that the capability of the PS system to potentially point to the trend of infections seemed to decrease with low participation rates. This finding is supported by Table 2, which reports the Pearson correlation coefficients between the PS and TS time series within each city.

Table 2 confirms that the largest correlation between the 2 data sources was indeed noted for the cities with the largest participation rates (namely, Teresina, Caruaru, and Santo André). The Pearson correlation coefficients for those 3 cities were above 0.5, indicating a moderate to high positive correlation between the 2 data sources.

We next analyzed the participation behavior of individuals across all 9 cities. A significant share of 40%-50% of individuals participated only once throughout the observation period, while roughly 6%-13% were considered as frequent participants, with at least 15 submitted reports. Detailed numbers can be found in Table S1 in Multimedia Appendix 1.

In order to assess the effect of lag on the time series correlation, we also determined the Pearson correlation coefficients for the 3 key cities with the largest public engagement, using 7-day and 14-day lags in the PS data series. Table 3 shows the lagged Pearson correlations for the full observation period, while Table 4 presents the results for the reduced period from April 1 to July 31, 2020.

In Table 3, an increased correlation can only be found for Teresina, where the coefficient increased from 0.82 to 0.89 for the 14-day lag. For Caruaru and Santo André, the coefficients declined from 0.79 to 0.71 and from 0.56 to 0.45, respectively. However, the correlations for the 7-day lag remained at roughly the same level. While these results are ambiguous and rather weak, the picture appears clearer in Table 4.

In Table 4, the coefficients remain either constant or show an increase using 7-day and 14-day lags across all 3 cities. By removing the tails of the observation period, the focus was set on the months with the strongest engagement, as shown by the higher daily average of submitted reports in Table 4.

Table 5 presents the proportion of confirmed COVID-19 cases, proportion of cases for which health care assistance was sought, and proportion of cases involving medication intake among all submitted reports that were categorized as potential COVID-19 cases.

**Table 1:** Participation ranking for the 9 Brazilian cities under examination.

City	Number of reports	Number of reports per 100^a^	Number of participants	Share of zeros^b^, %
Teresina	27,558	3.17	4449	7.0
Caruaru	10,279	2.81	1363	32.0
Santo Andre	14,207	1.97	2866	46.7
Niteroi	6757	1.31	897	62.9
Recife	3895	0.24	492	73.9
Porto Alegre	2779	0.19	336	96.1
Campinas	2006	0.17	307	92.9
Sao Paulo	12,453	0.10	2360	70.7
Rio de Janeiro	3071	0.05	734	74.6

^a^The ranking is based on the variable “number of reports per 100,” from the largest to the smallest. This variable shows the number of submitted reports per 100 inhabitants and is calculated as the number of submitted reports divided by population size times 100.

^b^The variable “share of zeros” shows the share of observation days on which no possible COVID-19 case was recorded within a city.

**Table 2:** Pearson correlations for each study city.

City^a^	Pearson correlation	*P* value
Teresina	0.82	<.001
Caruaru	0.79	<.001
Santo Andre	0.56	<.001
Niteroi	0.24	<.001
Recife	0.23	<.001
Porto Alegre	0.18	<.001
Campinas	−0.34	<.001
Sao Paulo	0.32	<.001
Rio de Janeiro	0.09	.19

^a^The cities are ranked according to population-weighted participation from the largest to the smallest.

**Table 3:** Lagged Pearson correlations for the full observation period (full 7-month period).

City	PC^a^	PC (7-day lag)	PC (14-day lag)	Average daily reports
Teresina	0.82	0.87	0.89	128
Caruaru	0.79	0.77	0.71	49
Santo Andre	0.56	0.56	0.45	66

^a^PC: Pearson correlation coefficient.

**Table 4:** Lagged Pearson correlations for the reduced observation period (April to July 2020).

City	PC^a^	PC (7-day lag)	PC (14-day lag)	Average daily reports
Teresina	0.86	0.91	0.93	193
Caruaru	0.85	0.85	0.88	76
Santo Andre	0.82	0.89	0.88	88

^a^PC: Pearson correlation coefficient.

**Table 5:** Characteristics of potential COVID-19 case reports.

Variable	Teresina (N=987), n (%)	Caruaru (N=356), n (%)	Santo Andre (N=189), n (%)
Confirmed cases	362 (36.7)	152 (42.7)	108 (57.1)
Cases seeking health care	270 (27.4)	112 (31.4)	65 (34.6)
Cases involving medication intake	495 (50.2)	185 (50.2)	110 (58.0)

### Forecasting Models

For the 3 cities with an at least moderate (higher than 0.8) positive Pearson correlation coefficient, we compared the RMSEs and MAEs from the baseline model with those from the combination as well as lagged combination model (Tables 6-8). We show the results for 1-day, 7-day, and 14-day forecasts.

Even though improvements were only modest, there was a pattern that could be identified across all 3 cities. The combination model outperformed the baseline model for the 14-day forecast by up to 2.7%. The results for the 7-day forecast were ambiguous; while there were improvements of up to 4.1%, only 2 out of the 3 cities showed reduced RMSEs.

**Table 6:** Forecasting errors using different models for 1-day, 7-day, and 14-day forecasts in the city of Teresina.

Forecast period and model^a^			RMSE^b^		MAE^c^	
**1-day** **forecast**						
	Baseline model	0.0195		0.0133		
	Combination model	0.0198		0.0140		
	Lagged combination model	0.0193		0.0137		
**7-day forecast**						
	Baseline model	0.0410		0.0302		
	Combination model	0.0393		0.0281		
	Lagged combination model	0.0409		0.0310		
**14-day** **forecast**						
	Baseline model	0.0347		0.0245		
	Combination model	0.0338		0.0246		
	Lagged combination model	0.0340		0.0249		

^a^The models used n=13 lagged components as independent variables.

^b^RMSE: root mean squared error.

^c^MAE: mean absolute error.

**Table 7:** Forecasting errors using different models for 1-day, 7-day, and 14-day forecasts in the city of Caruaru.

Forecast period and model^a^		RMSE^b^	MAE^c^
**1-day** **forecast**			
	Baseline model	0.0214	0.0162
	Combination model	0.0212	0.0160
	Lagged combination model	0.0290	0.0220
**7** **-day** **forecast**			
	Baseline model	0.0316	0.0234
	Combination model	0.0318	0.0237
	Lagged combination model	0.0329	0.0244
**14-day** **forecast**			
	Baseline model	0.0293	0.0190
	Combination model	0.0288	0.0187
	Lagged combination model	0.0292	0.0190

^a^The models used n=5 lagged components as independent variables.

^b^RMSE: root mean squared error.

^c^MAE: mean absolute error.

**Table 8:** Forecasting errors using different models for 1-day, 7-day, and 14-day forecasts in the city of Santo Andre.

Forecast period and model^a^		RMSE^b^	MAE^c^
**1-day** **forecast**			
	Baseline model	0.0137	0.0094
	Combination model	0.0236	0.0181
	Lagged combination model	0.0203	0.0157
**7** **-day** **forecast**			
	Baseline model	0.0225	0.0169
	Combination model	0.0219	0.0174
	Lagged combination model	0.0321	0.0245
**14-day** **forecast**			
	Baseline model	0.0292	0.0245
	Combination model	0.0284	0.0236
	Lagged combination model	0.0299	0.0251

^a^The models used n=14 lagged components as independent variables.

^b^RMSE: root mean squared error.

^c^MAE: mean absolute error.

## Discussion

The goal of this study was to show how PS systems, already well established as a routine surveillance monitoring approach for ILI, can also complement and enhance TS systems for monitoring pandemic diseases such as COVID-19. The results showed that the validity of the approach strongly depends on the rate of participation among the general population.

We found that the PS infection rates from the 3 cities with the largest participation approximately mirrored the TS infection rates, even though the representativeness of the PS subpopulation was most likely biased in terms of sex and age. The 3 cities were able to engage a large group of individuals due to social media campaigns promoted by local officials and governments. The insights from the other cities were less reliable as the data were not able to represent the traditional infection rates due to low engagement and zero inflation. In Campinas, the city with the lowest total number of submitted reports, PS performed so poorly that its data displayed a *negative* correlation with TS data. With a total of only 2006 submitted reports over a period of 210 days (Table 1), a daily average of self-reports of just under 10 was observed, implying that the sample size was far too small. The same was true for the other cities at the low end of the ranking, with Pearson correlations below 0.25. The only exception was São Paulo, whose correlation was smaller than the correlations of the top 3 cities. Even though it was ranked as second to last regarding population-weighted participation, the total number of submitted reports for São Paulo was relatively large at 12,453 reports. This corresponded to an average of nearly 60 submissions per day, resulting in a Pearson correlation coefficient of 0.32. Overall, low participation is considered one of the main limitations for the capability of PS data to mirror traditional infection rates. Even though it is known for ILI that participation of 1%-2% is reasonable in epidemic periods, additional work is necessary to better estimate a reasonable cutoff for COVID-19.

A stronger correlation between the TS and lagged PS time series supports the theory of early trend detection, which is one goal pursued by PS. However, this was only conducted for 3 cities, which showed a strong positive *PC_t_* above at least 0.5. It was found that toward the end of the observation period, only few people participated on the platform, such that around 80% of the reports were submitted within the period from April 1 to July 31, 2020.

The results from the forecasting analysis were also consistent with this conclusion. The errors for the 1-day ahead forecasts were generally smaller than the errors found in the 7-day and 14-day forecasts for all 3 cities. This is not surprising, as uncertainty rises with longer forecast horizons, and hence, the forecast accuracy of a model is lower. When looking at the 1-day forecasts, the baseline model seemed to outperform the combination model, at least for Teresina and Santo André. As mentioned before, uncertainty grows with longer horizons, which might explain the lack of value addition that comes from integrating PS data in a 1-day forecast. In Teresina, however, both the 7-day and 14-day forecasts from the combination and lagged combination models performed better than the baseline model, with slightly lower RMSEs. The combination model showed a 4.2% reduction in the RMSE for the 7-day horizon and a 2.6% reduction for the 14-day horizon, while the lagged combination model showed reductions of 0.2% and 2.1%, respectively. In Caruaru, the combination model performed slightly worse for the 7-day forecast relative to the baseline model, with a 0.5% increase in the RMSE, but an improvement was noted for the 14-day forecast, with a 1.7% reduction in the RMSE. Similar results were found for the lagged combination model, whereby the 7-day forecast showed a 4.1% increase in RMSE, while the 14-day forecast showed an improvement by 0.4%. The combination model applied on the data from Santo André again reduced the RMSEs for both the 7-day and 14-day forecasts by 2.8% and 2.7%, respectively. However, the lagged combination model was not able to improve the forecast for any of the applied horizons. This shows how complementing traditional disease surveillance systems may further increase the possibility for the early identification of outbreaks under the condition of sufficiently large participation. Slight improvements in the forecasting accuracy of up to 3% were identified for the models integrating data from both surveillance sources compared to the baseline model relying entirely on traditional data. Even if forecasting improvements are only weak, the detection of infections is improved as PS can cover an additional subset of the population that is overseen by the traditional system. The above findings contribute to a deeper understanding of the benefits of a complementary digital surveillance layer. They corroborate previous literature, emphasizing that the 2 approaches can be complements for timely health threat identification [3,14,16]. Even though some of the results indicate only small improvements in accuracy, the possibility of enhancing case detection through broader coverage cannot be neglected.

Some limitations related to the PS approach should be mentioned. Studies conducted in the United States and Western Europe suggest that the population subgroup captured by the *Brazil Sem Corona* PS system does not necessarily represent the general Brazilian population [31-33]. In previous PS data, female participants were significantly overrepresented compared to the general population. Besides that, age groups below 30 years and above 80 years were underrepresented. Moreover, the average participant most likely held a higher educational degree than the average population [34]. Furthermore, individuals living in bigger cities were more likely to participate, leading to clusters around more urban areas and information gaps in more remote regions. This is supported by the geocoordinates from the submitted reports on the *Brazil Sem Corona* platform, which indicate that a significant share of participants live in or around urban areas. Targeting the underrepresented population may prospectively improve the complementary benefit of the platform, particularly among those not seeking medical attention. Despite the biases found in the population covered by PS, studies highlight that PS systems that engage a sufficiently large group of participants can still adequately capture TS infection trends [33,35]. Ideally, PS tracks individuals throughout the season. Knowing that nearly half of the participants did not continue their engagement after the first report submission stresses the need for efforts to ensure more frequent participation in the future. Previous studies from the United States and Canada have found significant differences in participation across age groups, along with a 25% lower probability of frequent participation by women relative to men [33].

Another important aspect of complementing disease surveillance systems is the goal of early trend detection to identify disease outbreaks. Integrating multiple data sources not only aims at improving data insights and detecting more cases but also ideally leads to the detection of outbreaks at an earlier stage. The finding of greater coefficients in the lagged Pearson correlation indicates that the timeliness of the PS system helps in identifying slightly preceding trends. This supports the idea that a PS system, which engages a larger volunteer network, is more likely to depict infection trends, resulting in better data insights and, eventually, in earlier anticipation of trend changes. Recognizing outbreak patterns only slightly in advance might already have great benefits for health agencies when it comes to fighting pandemics. Improvements in 14-day forecasts allow health officials to respond more quickly and prioritize certain areas identified as more likely to suffer from rising infection numbers. This may be of particular importance for low-income countries or for regions that suffer from a considerable scarcity in health services. The PS system brings value by producing information that can be used to reduce uncertainty in allocation decisions. Furthermore, infection monitoring can be improved thanks to the geolocation information provided by PS data, which allows for a typically higher spatial resolution. Aside from preventing local transmissions, improvements in the surveillance system may result in externalities, such as lower infections in other regions [36].

We hypothesize that the severity of COVID-19 likely influenced individuals’ motivation to engage in a voluntary surveillance system. Governments have adjusted their behavior during the ongoing pandemic and made significant efforts to improve the timeliness of traditional data reporting. Many countries, including Brazil, have overcome administrative burdens and were ultimately able to report daily new infections. As such, the timeliness that usually distinguishes a PS system from a TS system has most likely vanished. We see this as a reasonable explanation for why the magnitude of the results from the forecasting is weaker compared to the findings of previous studies conducted in the field of ILI [14,15]. Besides that, ILI tracking allows for data collection across several flu seasons, increasing the amount of available data and allowing for consistency checks across seasons, whereas the novelty of COVID-19 only allows for a single 7-month observation period in Brazil.

We emphasize the successful collaboration among governments, locals, and professionals when it comes to maximizing the use and gain of the *Brazil Sem Corona* platform. The 3 cities with extensive social media campaigns clearly demonstrated the capabilities of the PS system, under the condition of sufficient participation. Defining sufficient participation remains relevant for future research; so far, previous studies only highlighted the number of reports as a critical element and claimed the need to maintain sufficient coverage without setting a certain threshold [13,32,37]. Factors, such as population density, urbanization, and area size, most likely cause regional variations, making it significantly more difficult to determine a specific number that is generally valid.

The implementation of a proper surveillance system remains an important challenge in developing countries. While the PS system aims to address disparities in health outcomes, it currently reaches more urban regions and relatively more educated people. Therefore, voluntary crowdsourced information likely contains a population bias. Assessing the potential benefits for more rural communities remains open to future research in order to realize the system’s full potential. Engaging and motivating a greater diversity of individuals remain key challenges that must be addressed in future PS platform implementations. Obstacles, such as lack of access to modern technologies, illiteracy, and simply lack of awareness of the benefits from participation, might hinder progress toward a more diverse reporting population.

Nearly half of the participants in Brazil submitted only a single report. This prevents monitoring the health status of individuals over a longer observation period, and as a result, reduces the insights that can be gained from PS systems. This stresses the need to evaluate incentives to induce more frequent participation. Additionally, in-depth research is needed to determine the reasons that lead to discontinuation of participation after the first submission. Addressing the above issues in future PS platform implementations is likely to lead to even greater benefits.

The unavailability of data on participants’ sociodemographic characteristics in the *Brazil Sem Corona* platform prevents an important analysis of the representativeness of participants versus the general Brazilian population. Performing further tests and extending the method could confirm the validity of the results and deepen the insights. The forecasting models were kept rather simple on purpose, with only the TS and PS data as independent variables. Adding mobility data or contact tracing data, for instance, could improve accuracy, leading to a better understanding of transmission. There are further limitations to keep in mind, such as the impact of the criteria of categorization of a submitted report into either a *suspected COVID-19* *case* or a *negative case,* as this can influence the number of reports included in the case counts and the likelihood of false positives. Furthermore, the applied local regression (“LOESS”) remains an approximation for smoothing the highly sparse data. Alternative smoothing techniques might produce different results, which could lead to divergent interpretations.

Public engagement remains a challenge. Future research needs to identify determinants of participation and proper incentives to induce larger coverage and higher diversity of participants. As the quality of data insights improves, PS benefits may further expand. Deeper insights allow for greater acceptance and credibility among governments and health authorities. Expanding collaboration among researchers, officials, and health authorities is needed to leverage data insights for timely response plans, which can ultimately lead to better health outcomes.

## Appendix

Multimedia Appendix 1 Data description.

## Abbreviations

ILI: influenza-like illness
MAE: mean absolute error
PS: participatory surveillance
RMSE: root mean squared error
TS: traditional surveillance

## Data Availability

The data that support the findings of this study are available from Colab, but restrictions apply to the availability of these data. The data were used under license for this study, and thus, they are not publicly available. The data are however available from the authors upon reasonable request and with permission from Colab.
