# Supplementary material for: Participatory Surveillance for COVID-19 Trend Detection in Brazil: Cross-sectional Study
Source: JMIR Public Health Surveill. 2023 Apr 26;9:e44517. doi: 10.2196/44517 (PMC10138922; doi:10.2196/44517)
Supplement: Multimedia Appendix 1 [file publichealth_v9i1e44517_app1.pdf]

Multimedia Appendix 1. Data description.

TS data description:

The traditional data report official lab confirmed daily new COVID-19 cases on a municipality level in Brazil. The data is publicly available and aggregated from sources such as the Ministério de Saude (Brazilian Ministry of Health) and Brasil.IO, a public data repository where epidemiological bulletins of each federative unit are compiled[22]. Across all nine cities, a total of 20 observation days indicate a negative amount of new cases due to corrections made for the preceding day. The TS infection ratio on these days is disregarded and dropped from the data set. For comparability reasons, the final data set only contains the observation days on which both the PS and TS infection rate is available.

PS data description:

Voluntary participants submitted self-reports on the *Brasil Sem Corona* platform through the Colab app available on mobile phones[24]. After the download, the terms and conditions had to be accepted andconsent to use the data for scientific purposes was given. Each user was allowed to submit a maximumof two reports a day and along every submitted report, information on latitude and longitude was gathered. For data protection reasons, the location information was automatically randomized in the server within a 2km radius. According to national privacy laws, users were able to drop out of the enrollment at any time, causing a complete deletion of the data from the server.

However, not all the submitted reports can be used for the analysis. Reports without either latitude or longitude information are disregarded, as they cannot be assigned to a municipality. There are some reports from outside the perimeter of Brazil, which are also ignored. Furthermore, users that are either detected as spammers, users that reported more than twice a day as well as users from the pilot study are dropped from the sample. This leads to a sample size of 127’809 reports over the time period of 7 month across all cities. Using QGIS, a geographic information system application that allows to analyze geospatial data, each report is then assigned to a municipality based on the longitudinal and

latitudinal information. Since the location is randomized within a radius of 2km, not only all the data points that lie within the border of a municipality are included, but also all those within a 2km buffer zone around the border. For the City of Teresina we include 266 additional data points that lie outside the 2km buffer zone, however, very closely to the border such that it can be associated to the same cluster. The argument is made that those participants are likely to be commuters and thus equally exposed to the advertisement campaign in Teresina. SI Figure 1 shows the daily number of submitted reports over the observation period March until October 2020.

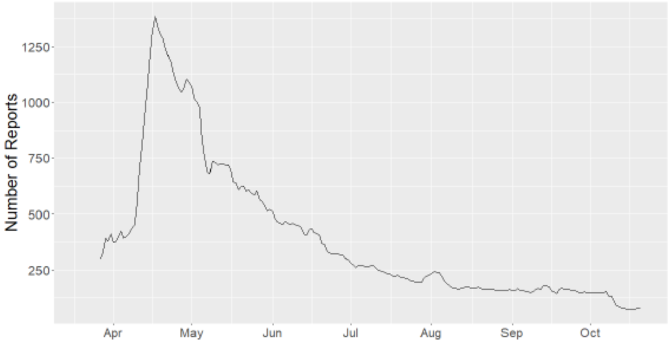

SI Figure 1: Daily number of submitted reports This figure shows a 7-day rolling average of submitted reports from participants across all cities observed.

Due to the nature of the data, the daily rates are highly volatile and thus, must be smoothed. Therefore, we take advantage of a powerful but simple approach to fit smooth lines to empirical data called Loess. Loess is a commonly used method for local regressions. It uses a non-parametric approach, meaning that there is no need for a priori specifications of the relationship [30]. Only the proportion of observations that is used in each local regression needs to be specified. This parameter is also called alpha and lies between 0 and 1. We set alpha equal to 0.3 in order to get rid of the volatility while still allowing for peaks and valleys in the data.

To test for non-stationary behavior, we use the Augmented Dickey-fuller test. Since the p-value is found to be greater than the threshold value of 0.05, we cannot reject the null-hypothesis stating that the data is non-stationary. To avoid inconsistent and unreliable results, we transform both the TS and PS variables into stationary data by applying second-order differencing. Additionally, we drop those

observations which lie outside the range of +/- 3 standard deviation around the mean to remove outliers. For the City of Caruaru, we decided to remove observations outside the range of +/- 2 standard deviations around the mean as the data showed greater volatility.

Supporting the main data analysis, we additionally analyze the reporting behavior of the *Brasil Sem Corona* participants. We find that up to 50% of the users only submitted a single report throughout the observation period, while 7% - 14% participated on a regular basis with at least 15 submissions.

City	One-time Participation	Frequent Participation
Teresina	42%	10%
Caruaru	39%	12%
Santo Andre	48%	7%
Niteroi	39%	13%
Recife	40%	12%
Porto Alegre	49%	14%
Campinas	43%	11%
Sao Paulo	50%	8%
Rio de Janeiro	49%	6%

SI Table 1: Participation Behavior This table shows the share of individuals that participated only a single time (One-time Participation) and of those that submitted reports at least 15 times (Frequent Participation).

To check for the robustness in the forecasting results, we adjust the training and test sample split to a 70% / 30% as well as a 90% / 10% split. As the number of observations included in the model fitting process changes with the split size, the forecast errors slightly change. However, the overall picture remains consistent. Both for the 70% / 30% and the 90% / 10% split the Combination model shows slightly higher accuracy for the 14-day horizon. The magnitude of the increased accuracy remains on a similar level, between 1.3% and 3.5%. The detailed results can be found below in SI Table 2.

A: Teresina

Forecast period	Model	RMSE	MAE
Forecast t+1	Baseline model	0.0200	0.0142
	Combination model	0.0228	0.0176
	Lagged Combination model	0.0207	0.0153
Forecast t+7	Baseline model	0.0350	0.0252
	Combination model	0.0349	0.0251
	Lagged Combination model	0.0364	0.0267
Forecast t+14	Baseline model	0.0317	0.0230
	Combination model	0.0313	0.0226
	Lagged Combination model	0.0315	0.0238

Note: The model used n=13 lagged components as independent variables

B: Caruaru

Forecast period	Model	RMSE	MAE
Forecast t+1	Baseline model	0.0188	0.0139
	Combination model	0.0208	0.0148
	Lagged Combination model	0.0244	0.0179
Forecast t+7	Baseline model	0.0332	0.0254
	Combination model	0.0328	0.0249
	Lagged Combination model	0.0352	0.0273
Forecast t+14	Baseline model	0.0324	0.0230
	Combination model	0.0318	0.0222
	Lagged Combination model	0.0327	0.0237

Note: The model used n=5 lagged components as independent variables

C: Santo Andre

Forecast period	Model	RMSE	MAE
Forecast t+1	Baseline model	0.0125	0.0086
	Combination model	0.164	0.0117
	Lagged Combination model	0.0175	0.0118
Forecast t+7	Baseline model	0.0200	0.0142
	Combination model	0.0218	0.0167
	Lagged Combination model	0.0217	0.0163
Forecast t+14	Baseline model	0.0226	0.0170
	Combination model	0.0221	0.0165
	Lagged Combination model	0.0230	0.0177

Note: The model used n=5 lagged components as independent variables

A: Teresina

Forecast period	Model	RMSE	MAE
Forecast t+1	Baseline model	0.0243	0.0158
	Combination model	0.0230	0.0157
	Lagged Combination model	0.0232	0.0155
Forecast t+7	Baseline model	0.0535	0.0414
	Combination model	0.0507	0.0377
	Lagged Combination model	0.0531	0.0414
Forecast t+14	Baseline model	0.0316	0.0201
	Combination model	0.0306	0.0210
	Lagged Combination model	0.0291	0.0193

Note: The model used n=13 lagged components as independent variables

B: Caruaru

Forecast period	Model	RMSE	MAE
Forecast t+1	Baseline model	0.0261	0.0200
	Combination model	0.0272	0.0203
	Lagged Combination model	0.0289	0.0228
Forecast t+7	Baseline model	0.0371	0.0265
	Combination model	0.0353	0.0257
	Lagged Combination model	0.0383	0.0280
Forecast t+14	Baseline model	0.0150	0.0100
	Combination model	0.0145	0.0096
	Lagged Combination model	0.0154	0.0107

Note: The model used n=5 lagged components as independent variables

C: Santo Andre

Forecast period	Model	RMSE	MAE
Forecast t+1	Baseline model	0.0167	0.0125
	Combination model	0.0122	0.0103
	Lagged Combination model	0.0202	0.0152
Forecast t+7	Baseline model	0.0271	0.0215
	Combination model	0.0237	0.0188
	Lagged Combination model	0.0344	0.0281
Forecast t+14	Baseline model	0.0370	0.0335
	Combination model	0.0380	0.0345
	Lagged Combination model	0.0389	0.0354

Note: The model used n=14 lagged components as independent variables

SI Table 2: Forecast errors: Left: 70% / 30% data split. Right: 90% / 10% data split. **A**: Displays the forecasting errors for the city of Teresina using a Baseline model, Combination model and Lagged Combination model. Errors are calculated for a one-day, seven-day and 14-day horizon. **B**: Similar to Panel A, it shows the results for the city of Caruaru. **C**: Similar to Panel A, it shows the results for the city of Santo Andre.

References

22. Cota, W. Monitoring the number of COVID-19 cases and deaths in Brazil at municipal and federative units level. *SciELOPreprints:362*; 10.1590/scielopreprints.362 (2020).

24. Brasil Sem Corona. Brasil Sem Corona Participatory Surveillance Platform. Available at https://www.brasilsemcorona.com.br/ (2021).

30. Jacoby, W. G. Loess: a nonparametric, graphical tool for depicting relationships between variables. *Electoral Studies* **19,** 577–613; 10.1016/S0261-3794(99)00028-1 (2000).
